# Sepsis induces muscle atrophy by inhibiting proliferation and promoting apoptosis via PLK1‐AKT signalling

**DOI:** 10.1111/jcmm.16921

**Published:** 2021-09-12

**Authors:** Ying‐Ya Cao, Zhen Wang, Tao Yu, Yuan Zhang, Zhong‐Han Wang, Zi‐Meng Lu, Wei‐Hua Lu, Jian‐Bo Yu

**Affiliations:** ^1^ Department of Anesthesiology and Critical Care Medicine Tianjin Nankai Hospital Tianjin Medical University Tianjin China; ^2^ Department of Intensive Care Unit The First Affiliated Hospital of Wannan Medical College Wuhu Anhui China; ^3^ Department of Neurosurgery The First Affiliated Hospital of Wannan Medical College Wuhu Anhui China; ^4^ College of Food Science and Engineering Northwest A&F University Yangling Shanxi China

**Keywords:** apoptosis, muscle atrophy, polo‐like kinase 1, proliferation, sepsis

## Abstract

Sepsis and sepsis‐induced skeletal muscle atrophy are common in patients in intensive care units with high mortality, while the mechanisms are controversial and complicated. In the present study, the atrophy of skeletal muscle was evaluated in sepsis mouse model as well as the apoptosis of muscle fibres. Sepsis induced atrophy of skeletal muscle and apoptosis of myofibres in vivo and in vitro. In cell‐based in vitro experiments, lipopolysaccharide (LPS) stimulation also inhibited the proliferation of myoblasts. At the molecular level, the expression of polo‐like kinase 1 (PLK1) and phosphorylated protein kinase B (p‐AKT) was decreased. Overexpression of PLK1 partly rescued LPS‐induced apoptosis, proliferation suppression and atrophy in C2C12 cells. Furthermore, inhibiting the AKT pathway deteriorated LPS‐induced atrophy in PLK1‐overexpressing C2C12 myotubes. PLK1 was found to participate in regulating apoptosis and E3 ubiquitin ligase activity in C2C12 cells. Taken together, these results indicate that sepsis induces skeletal muscle atrophy by promoting apoptosis of muscle fibres and inhibiting proliferation of myoblasts via regulation of the PLK1‐AKT pathway. These findings enhance understanding of the mechanism of sepsis‐induced skeletal muscle atrophy.

## 1 INTRODUCTION

Skeletal muscle atrophy, which is defined as a decrease in muscle mass that leads to weakness, is one of the complications of sepsis that prolongs intensive care unit (ICU) treatment time and worsens long‐term patient outcomes.^1^, ^2^, ^3^ The muscles in septic patients present various signs of bioenergetic failure, including ATP depletion, mitochondrial dysfunction and oxidative stress, which ultimately lead to a reduction in muscle mass.^4^

Skeletal muscle atrophy is regarded as the result of an imbalance between myofibrillar protein synthesis and degradation.^5^, ^6^ Ubiquitin‐proteasome system (UPS)‐mediated proteolysis, autophagy and the calcium‐dependent calpain pathway are known as the three pathways that regulate protein degradation in muscle, among which UPS‐mediated proteolysis plays a prominent role in skeletal muscle atrophy.^7^ Muscle ring finger protein 1 (MuRF1) and atrophy gene‐1 (Atrogin‐1), two UPS enzyme proteins that are widely used as sensitive markers of skeletal muscle atrophy, are critical E3 ubiquitin ligases that mediate proteolysis in muscles and remain highly expressed during skeletal muscle atrophy.^8^, ^9^

The mechanism of sepsis‐induced skeletal muscle atrophy has been widely investigated in recent years. From an ultrastructural perspective, sepsis decreases the activity of complex I and complex IV, thus decreasing the ATP/ADP ratio, impairing mitochondrial respiration and elevating the production of reactive oxygen species (ROS); this mechanism results in mitochondrial dysfunction in skeletal muscles.^10^, ^11^, ^12^ On the other hand, sepsis increases the serum levels of proinflammatory cytokines such as tumour necrosis factor alpha (TNF‐α) and interleukin‐6 (IL‐6) and increases microvascular permeability. The entrance of toxins into the circulation can impair axon activity, thus reducing the nutrient supply to muscle and leading to muscle atrophy.^13^ Although numerous studies have been performed, little attention has been given to the proliferation and apoptosis of muscle cells. Previous studies have shown that apoptosis regulates the number of muscle cells and that muscle cell apoptosis results in skeletal muscle atrophy and sarcopenia in humans.^14^, ^15^ Nevertheless, the mechanism is not yet clear.

Polo‐like kinase 1 (PLK1), a member of the polo‐like kinase family, is a cell cycle‐related kinase that is required for proper M‐phase progression. PLK1 inhibition leads to pronounced mitotic arrest, followed by apoptosis.^16^, ^17^ The protein kinase B (AKT) signalling pathway is involved in the regulation of apoptosis, proliferation and metabolism in skeletal muscle.^18^ Activation of the AKT pathway is sufficient to block skeletal muscle atrophy.^19^ Notably, previous studies have indicated that PLK1 is an upstream kinase of AKT.^20^, ^21^

Here, we hypothesized that sepsis induces apoptosis and inhibits the proliferation of muscle cells, thus resulting in muscle atrophy, and this process is regulated by PLK1‐AKT signalling. To verify this hypothesis, a sepsis‐induced muscle atrophy model was established in vivo and in vitro, and then, the possible mechanisms were explored. Our findings further promote the understanding of sepsis‐induced skeletal muscle atrophy.

## 2 MATERIALS AND METHODS

### 2.1 Sepsis model

Caecal ligation and perforation (CLP) was performed on 8‐week‐old male C57BL/6 mice weighing 25–28 g purchased from Keygen Biotech (Nanjing, China) to establish a sepsis model as described previously.^22^ The mice were housed in the SPF animal laboratory with free access to diet and water for at least l week before use. Briefly, mice were weighed and anaesthetized with pentobarbital (50 mg/kg). Then, a midline laparotomy was performed, and the caecum was removed. The caecum was ligated at the distal 3/4 of the caecum, followed by a single ‘through and through’ perforation (21‐gauge needle). After puncture, the caecum was returned to the abdomen, and then, the incision was sutured. The mice in the sham group underwent laparotomy without CLP. Saline solution was injected subcutaneously (50 mg/kg) for resuscitation after the surgery. All animal experiments were performed according to the National Institutes of Health Guide for the Care and Use of Laboratory Animals. The animal experiments were authorized by the Institutional Animal Care and Use Committee of Wannan Medical College.

### 2.2 Weight measurement

The weight of the gastrocnemius was interpreted as an evaluation of muscle mass loss. The skin of the hind limbs was stripped, and the gastrocnemius of mice was collected and weighed at baseline and at 72 hours after CLP.

### 2.3 Tissue histology

Mice were sacrificed 72 hours after CLP, and gastrocnemius and soleus tissues were dissected, fixed with 10% buffered formalin overnight at room temperature, embedded in paraffin and sectioned into 4‐µm thick sections. Subsequently, haematoxylin and eosin (H&E) was used to stain the sections, and optical microscopy was used for morphological evaluation. The cross‐sectional area (CSA) of myofibres was recorded to evaluate the muscle fibre size.

### 2.4 TUNEL assay

The TUNEL assay (Roche, Mannheim, Germany) was performed to detect apoptotic cells in the paraffin‐embedded tissue sections according to the manufacturer's protocol. The positive cells were counted at 4–6 different areas per section and reported as a percentage of TUNEL‐positive muscle cells.

### 2.5 Cell culture and treatment

C2C12 myoblasts were purchased from Jiangsu Keygen Biotech Corporation (Nanjing, China). The cells were cultured in DMEM (Gibco, NY, USA) supplemented with 10% (v/v) FBS (Gibco), penicillin (100 U/ml) and streptomycin (100 mg/ml) at 37°C and 5% CO_2_ in a humidified incubator. To induce differentiation, cells were cultured until they reached 80% confluence and were then cultured for 5 days in differentiation medium (DM) consisting of DMEM supplemented with 2% horse serum (Gibco). Then, C2C12 myotubes were incubated with lipopolysaccharide (LPS, Sigma, L2880, 055:B5) at the indicated concentrations and for the indicated times. Vehicle (DMEM)‐treated cells were used as controls.

### 2.6 Giemsa staining

After the indicated treatments, C2C12 myotubes were washed with PBS, fixed in absolute methanol for 10 min and incubated with 10% Giemsa reagent for 10 min at room temperature. Subsequently, myotubes were washed with PBS three times and visualized using light microscopy.

### 2.7 C2C12 myotube diameter measurement

The diameter of C2C12 myotubes was determined using a digital camera mounted on an electron microscope. Briefly, we randomly chose 5 myotubes per field, and then, each myotube was measured along the length at three points in a blinded fashion. The diameter per myotube was expressed as the mean of three measurements, and at least 50 myotubes were measured.

### 2.8 Immunofluorescence staining

The expression of myofibre‐specific myosin heavy chain (MyHC), which is a morphological parameter of muscle differentiation,^23^ was detected in myotubes with immunofluorescence staining. Briefly, the treated C2C12 myotubes were fixed in 4% paraformaldehyde for 15 min, washed with PBS 3 times, incubated with Triton X‐100 and blocked with 5% (v/v) normal goat serum as previously described.^24^ An anti‐MyHC antibody (1:200 dilution, Abcam) was used for staining, and images were acquired using a confocal laser scanning microscope (Olympus).

### 2.9 Apoptosis detection

Apoptotic cells were double‐labelled with Annexin V‐fluorescein isothiocyanate (FITC) and 7‐amino‐actinomycin D (7‐AAD) using an Annexin V‐FITC/7‐AAD kit (Neo Bioscience) and were analysed by flow cytometry. Briefly, the induced myotubes were exposed to the indicated concentration of LPS for 24 h. After treatment, both floating and attached cells were collected, washed, resuspended in PBS and then stained with Annexin V and 7‐AAD solutions. The percentage of Annexin V‐positive cells was calculated. The experiment was repeated three times, and the results are presented as the mean values.

### 2.10 Cell viability assay

The effects of LPS on C2C12 myoblast viability were assessed using a Cell Counting Kit‐8 (CCK‐8, Dojindo, Japan) assay as previously described.^25^ Briefly, the myoblasts were plated in 96‐well plates at a density of 1 × 10^4^ cells per well, and after treatment with LPS at the indicated concentrations and for the indicated times, CCK‐8 (10 μl) was added to each well and incubated at 37°C for 1 h. Absorbance (450 nm) was measured using a microplate spectrophotometer.

### 2.11 5‐Ethynyl‐2’‐deoxyuridine (Edu) Assays

An EdU assay kit (Keygen Bio) was used to determine the cell proliferation rate according to the manufacturer's instructions. Briefly, C2C12 myoblasts with the indicated treatments were incubated at 37°C in EdU solution for 2 h and then fixed in 4% formaldehyde for 30 min at room temperature. The cells were then incubated with 100 μl of Apollo^®^ reagent for 30 min, and the nuclei were stained with Hoechst 33342 for 30 min. The fluorescence intensity was captured by fluorescence microscopy (Eclipse Ti‐U, Nikon).

### 2.12 Small interfering RNA (siRNA) and plasmid constructs

The following target sequence of PLK1 (GenBank accession no. NM_011121) was used to silence PLK1: 5′‐AGATCACCCTCCTTAAATATT‐3′. The scrambled siRNA sequence 5′‐TTCTCCGAACGTGTCACGT‐3′ was used as a negative control. The oligonucleotides were chemically synthesized by Gene Pharma (Shanghai, China). The full‐length PLK1 coding region was amplified from total cDNA using the forward primer 5’‐CCGCTCGAGGGAGATGAGTGCTGCAGTGAC‐3’, which contained an XhoI site, and the reverse primer 5’‐CCGGAATTCCTATTAGGAGGCCTTGAGACGG‐3’, which contained an EcoRI site. The amplified sequence was inserted into pcDNA 3.1 to generate pcDNA‐PLK1‐myc. Successful plasmid construction was confirmed by DNA sequencing.

### 2.13 Transfection with siRNA and plasmids

C2C12 myoblasts were seeded in 6‐well plates with DMEM containing 10% FBS. When the cells reached 60–70% confluence, the media were changed to Opti‐MEM reduced serum medium (Gibco) before transfection. Then, equal amounts of the siRNA/plasmid and control were transfected into myoblasts with Lipofectamine 3000 (Invitrogen) according to the manufacturer's instructions.

### 2.14 Western blot analysis

Western blotting was used to measure the cellular protein levels. Cells were washed with cold PBS and then lysed in radioimmunoprecipitation assay lysis buffer containing protease inhibitor and phosphatase inhibitor cocktails. Total protein concentrations were measured using a protein assay kit (Bio‐Rad). Equal amounts of protein samples were separated by 10% sodium dodecyl sulphate‐polyacrylamide gel electrophoresis (SDS‐PAGE). Then, the resolved proteins were transferred onto polyvinylidene fluoride (PVDF) membranes (Millipore, Bedford, MA, USA) at 100 V for 1 h at 4°C. Subsequently, the membranes were blocked with Tris‐buffered saline‐Tween (TBST) containing 5% nonfat dry milk for 1 h at room temperature. After being blocked, the membranes were probed with the indicated primary antibody overnight at 4°C and then blotted with the respective secondary antibodies. The membranes were analysed using super ECL detection reagent (Applygen, Beijing, China). The details of the primary antibodies used in the study were shown in Table 1.

**TABLE 1:** The details of primary antibodies for Western blotting

Antibody	Manufacturer	Catalogue numbers	Dilution
anti‐PLK1	Upstate	05‐844	1:1000
anti‐total‐Caspase3	Abcam	ab184787	1:1000
anti‐cleaved‐Caspase 3	Cell Signaling Technology	9664s	1:500
anti‐p‐AKT	Cell Signaling Technology	4060s	1:500
anti‐AKT	Cell Signaling Technology	9272s	1:500
anti‐MuRF1	Abcam	ab201941	1:500
anti‐Atrogin‐1	Abcam	ab168372	1:500
anti‐Myc	Santa Cruz Biotechnology	sc‐40	1:500
anti‐p‐mTOR	Abcam	ab109268	1:1000
anti‐mTOR	Abcam	ab134903	1:1000
anti‐p‐S6K	Abcam	ab59208	1:1000
anti‐ S6K	Abcam	ab186753	1:1000
anti‐p‐4EBP1	Abcam	ab259329	1:1000
anti‐4EBP1	Abcam	ab32024	1:1000
anti‐β‐actin	Proteintech	20536‐1‐AP	1:1000

### 2.15 RNA isolation and quantitative real‐time polymerase chain reaction (qRT‐PCR)

The whole gastrocnemius tissue total RNA content was used as a measure of ribosome abundance and translation capacity. Total RNA was extracted from muscle tissues of equal quality using TRIzol reagent (Invitrogen) according to the manufacturer's instructions, and the whole‐tissue RNA content was calculated by multiplying the total RNA concentration by the muscle weight. Total RNA from cultured cells was extracted using a similar method. cDNA synthesis was performed using a Prime Script™ RT Reagent Kit (Takara) according to the manufacturer's instructions, and qRT‐PCR analysis was subsequently performed using a SYBR PrimeScript mRNA real‐time PCR kit (Takara) according to the manufacturer's protocol. The primer sequences used in the study were shown in Table 2.

**TABLE 2:** The primer sequences for RT‐qPCR

Gene	Forward primer	Reverse primer
MuRF1	5’‐ CGACATCTTCCAGGCTGCGAAT−3’	5’‐ATCACTTCATGGCGGCACGAG−3’
Atrogin−1	5’‐CCATTCTACACTGGCAGCAGCA−3’	5’‐ ACAGGCAGGTCGGTGATCGT−3’
28S rRNA	5’‐GCCATGGTAATCCTGCTCAGTAC−3’	5’‐ GCTCCTCAGCCAAGCACATAC−3’
18S rRNA	5’‐CGGACCAGAGCGAAAGCA−3’	5’‐ACCTCCGACTTTCGTTCTTGATT−3’
GAPDH	5’‐ AAGGTCGGTGTGAACGGATT−3’	5’‐ TGAGTGGAGTCATACTGGAACAT−3’
HRPT	5’‐ TACAGGCCAGACTTTGTTGG−3’	5’‐ TTGGCTTTTCCACTTTCGCTG−3’

### 2.16 Statistical analysis

The data are expressed as the means ±SD of 3 independent experiments. The experimental results were statistically evaluated using Student's *t* tests or one‐way ANOVA. Survival was analysed by log‐rank test. For all statistical tests, PRISM 5.0 (GraphPad Software Inc., San Diego, CA) was used. *p* values <0.05 were considered statistically significant.

## 3 RESULTS

### 3.1 Sepsis‐induced skeletal muscle atrophy in mice

**FIGURE 1:**
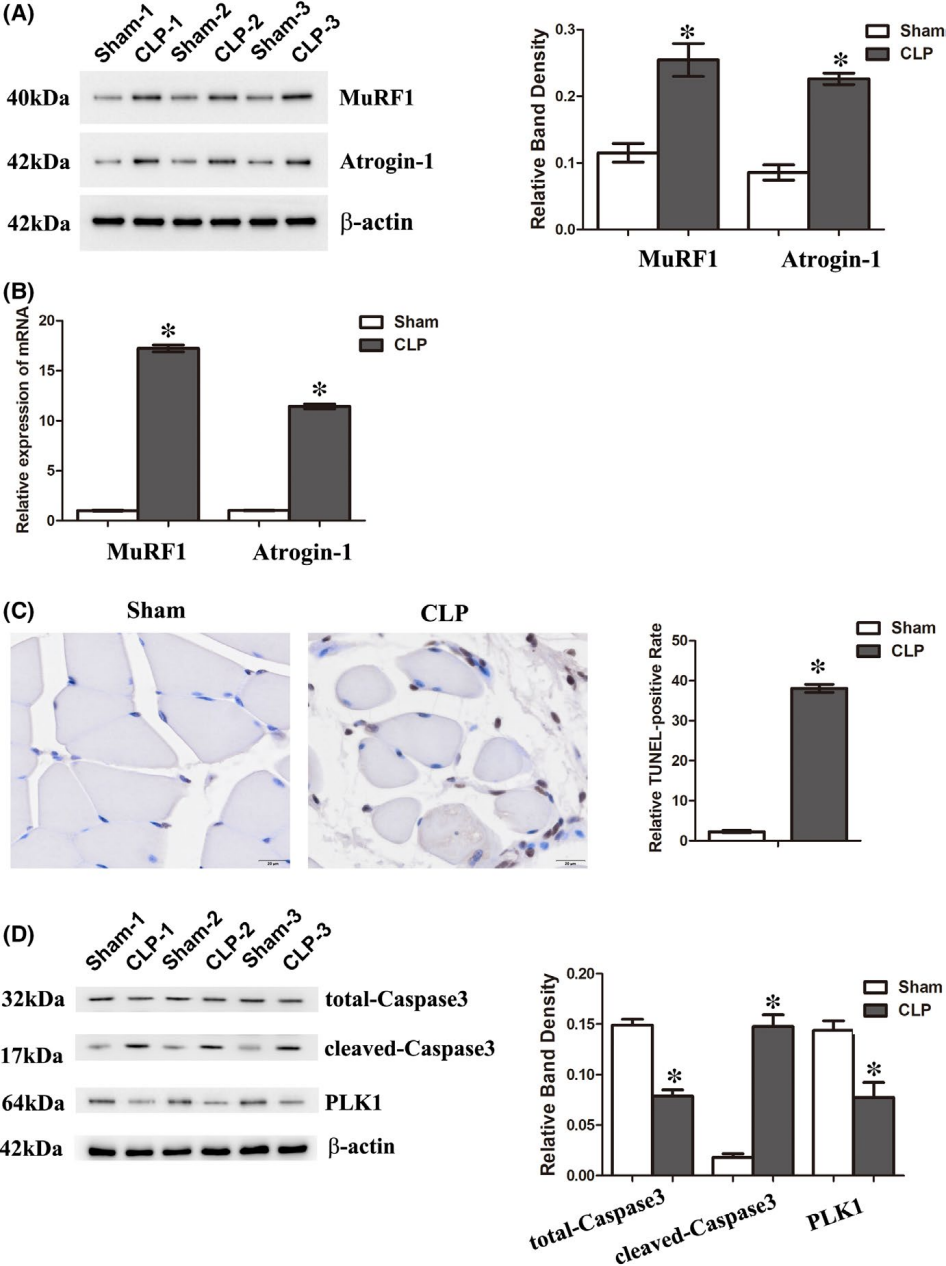
Sepsis‐induced atrophy and apoptosis of gastrocnemius myofibres accompanied with downregulation of PLK1 in mice. Mice underwent CLP to establish a sepsis model and were sacrificed 3 days later. The gastrocnemius was extracted and analysed. (A) The expression of MuRF1 and Atrogin‐1 was measured by Western blotting. The graph shows the relative band densities. (B) The mRNA levels of MuRF1 and Atrogin‐1 were measured by qRT‐PCR. (C) A TUNEL assay was used to detect the apoptotic gastrocnemius cells in each group. The percentage of positive cells was analysed. Scale bar =20 µm. (D) The expression of the indicated proteins was measured by Western blotting. The graph shows the relative band densities. Each result was replicated in three independent experiments, and the values are the means ±SD. *p < 0.05 versus the control group (n = 3)

The mortality of the mice was evaluated in the 72‐hour follow‐up period (Figure S1A). To evaluate the effect of sepsis on skeletal muscle atrophy, the gastrocnemius and soleus were analysed in the CLP mouse model. H&E staining and CSA measurement were used to assess the pathological changes associated with muscle atrophy (Figure S1C‐D). The gastrocnemius and soleus muscles of septic mice exhibited disorder and aneuros myofibres with reduced CSAs. Furthermore, the weight of the gastrocnemius muscles of septic mice significantly decreased (Figure S1B). We also measured the expression of MuRF1 and Atrogin‐1, two sensitive markers of muscular atrophy. The Western blot and qRT‐PCR results showed that MuRF1 and Atrogin‐1 levels were significantly increased in septic mice (Figure 1A‐B). Considering that a decrease in protein synthesis can contribute to muscle atrophy, translation efficiency and capacity were assessed by measuring the expression of p‐mTOR, p‐S6K and p‐4EBP1 and the levels of total RNA, 18S and 28S ribosomal RNA. The results revealed decreased p‐mTOR, p‐S6K and p‐4EBP1 expression and reduced total RNA and 18S and 28S ribosomal RNA levels in the gastrocnemius tissues of septic mice (Figure S2A‐D). These data indicated that skeletal muscle atrophy occurred in the septic mice.

### 3.2 Sepsis‐induced apoptosis of myofibres

Apoptosis plays an important role in skeletal muscle atrophy.^26^ In our study, a TUNEL assay was applied to identify apoptotic nuclei in muscle fibres. Compared to the control group, the number of TUNEL‐positive nuclei per section of gastrocnemius muscle fibres increased significantly in the sepsis group after 3 days of CLP (Figure 1C). The expression of total‐Caspase3 and cleaved‐Caspase3 was also determined to assess the apoptosis of myofibres (Figure 1D). The levels of total‐Caspase3 were decreased in the sepsis group, while those of cleaved‐Caspase3 were elevated; these findings were consistent with the results of the TUNEL assay and suggested that apoptosis of myofibres occurred during sepsis.

### 3.3 Sepsis downregulated PLK1 expression of myofibers

A previous study revealed that PLK1 plays a critical role in developmental and regenerative myogenesis, while its function in myofiber atrophy is still unknown.^27^ Here, the expression of PLK1 was detected in sepsis‐induced atrophic gastrocnemius, and the results showed that PLK1 was reduced in atrophic gastrocnemius (Figure 1D).

### 3.4 LPS‐induced atrophy of C2C12 myotubes

**FIGURE 2:**
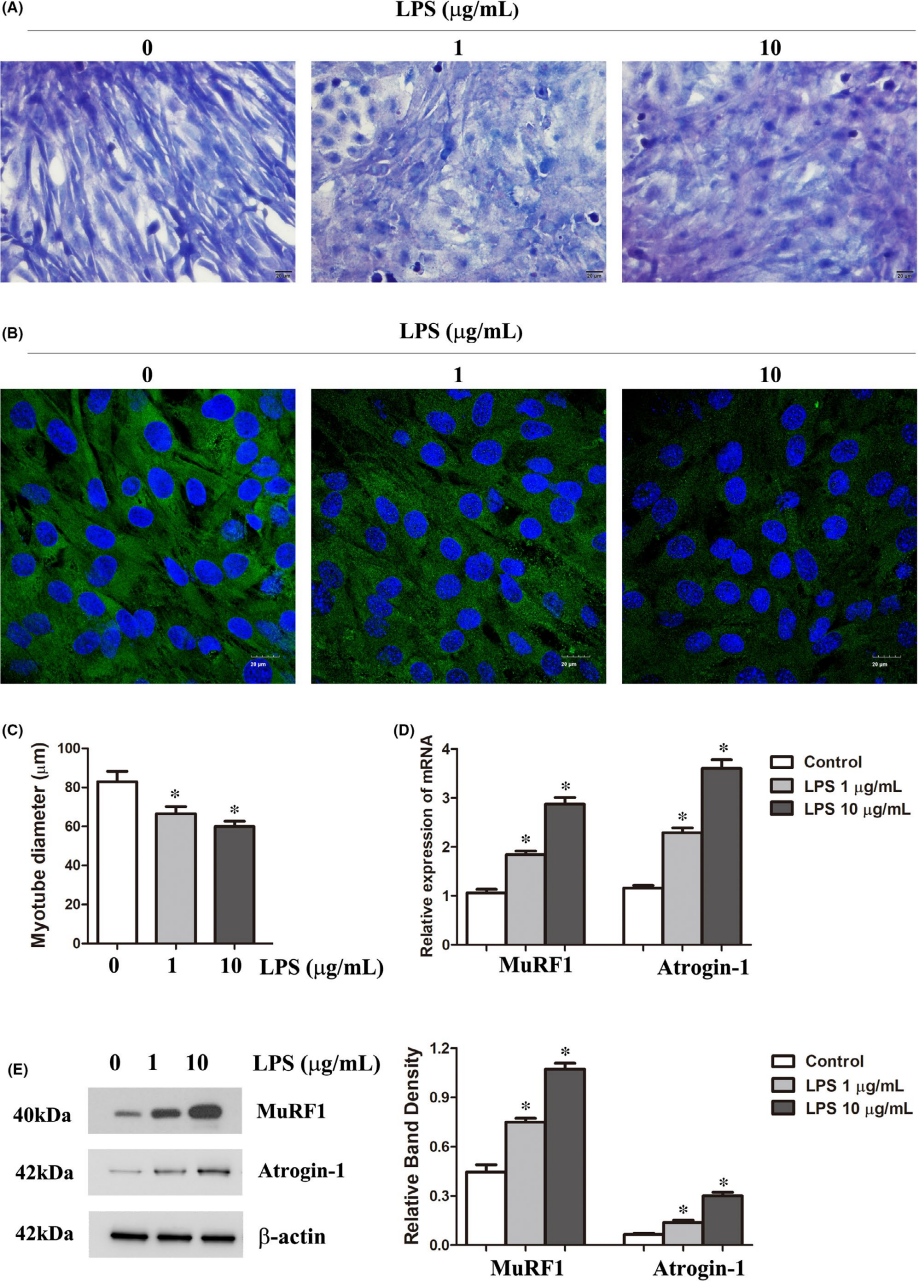
LPS‐induced atrophy of C2C12 myotubes. Myotubes were incubated with 0, 1 or 10 μg/ml LPS for 24 h. (A) Treated and untreated myotubes were stained with Giemsa reagent, and bright field images were taken at the same magnification. Scale bar =20 µm. (B) Representative immunofluorescence staining of MyHC in C2C12 myotubes treated as indicated for 24 h. Scale bar =20 µm. (C) The diameters of the myotubes after the indicated treatments for 24 h. (D) Quantitative RT‐PCR analysis of MuRF1 and Atrogin‐1 levels in C2C12 myotubes after the indicated treatments for 24 h. (F) The protein expression levels of MuRF1 and Atrogin‐1 in C2C12 myotubes after the indicated treatments for 24 h. Each result was replicated in three independent experiments, and the values are the means ±SD. *p < 0.05 versus the control group

To further investigate the role of PLK1 in sepsis‐induced muscle atrophy, we established a sepsis model in vitro using C2C12 cells. First, the effect of LPS on C2C12 myotube atrophy was verified by using Giemsa staining to observe the morphology of the myotubes. After LPS treatment, we observed a morphological reduction in myotube size (Figure 2A). The expression of MyHC was also measured to evaluate the abundance of mature myotubes, and the results indicated that MyHC protein expression clearly decreased with LPS treatment (Figure 2B), which was consistent with the findings of a previous study.^28^ Then, we measured the diameters of C2C12 myotubes and found that the diameters were decreased by LPS treatment (Figure 2C). Furthermore, we measured the expression of MuRF1 and Atrogin‐1 in LPS‐treated C2C12 myotubes. The qRT‐PCR and Western blot results showed that MuRF1 and Atrogin‐1 levels were increased in LPS‐treated C2C12 myotubes (Figure 2D‐E), indicating atrophy of C2C12 myotubes.

### 3.5 LPS promoted apoptosis in C2C12 myotubes

**FIGURE 3:**
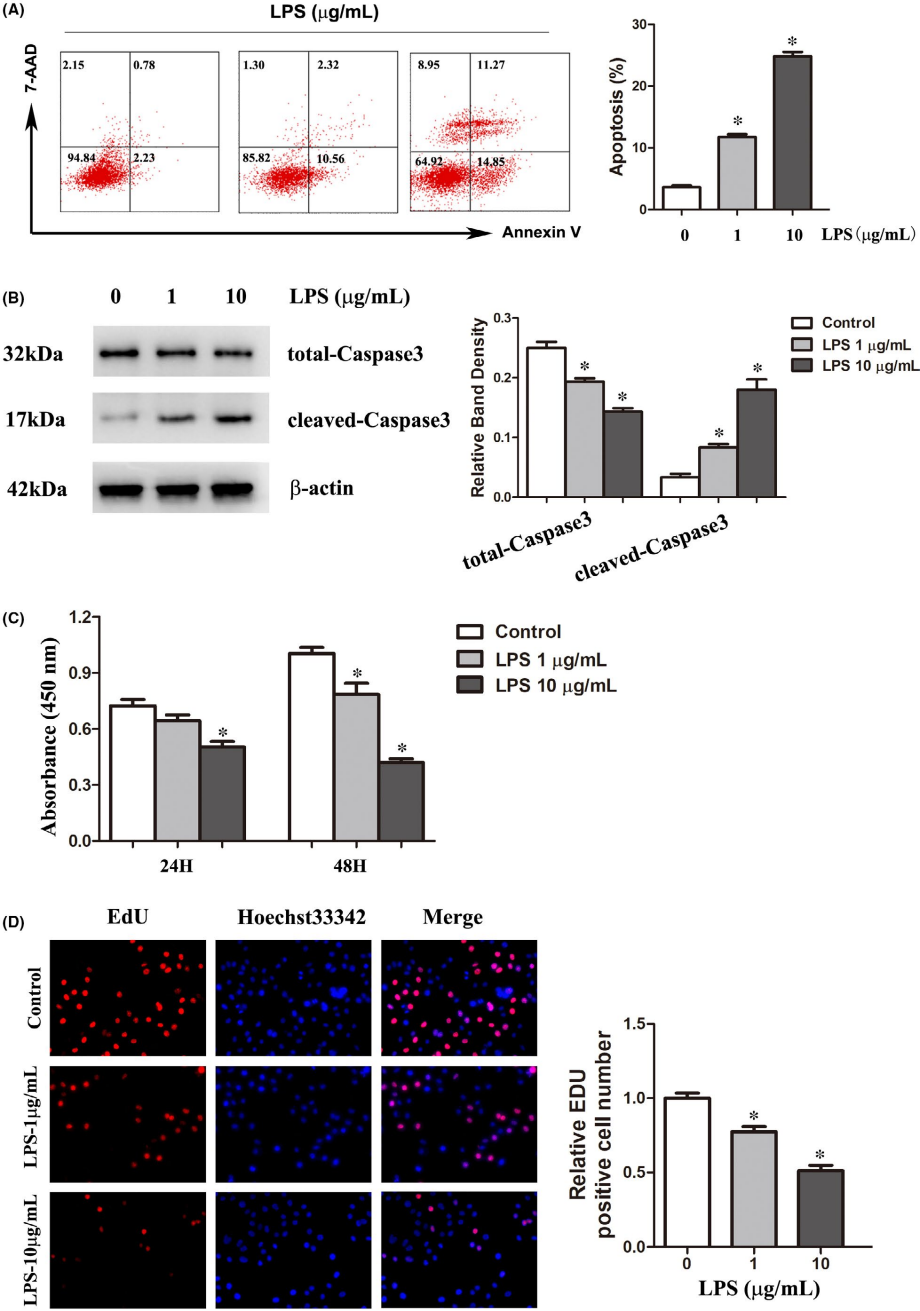
LPS promoted apoptosis in C2C12 myotubes and inhibited the proliferation of C2C12 myoblasts. (A) C2C12 myotubes were incubated with 0, 1 or 10 μg/ml LPS for 24 h. Apoptosis was analysed by Annexin V‐FITC/7‐AAD double‐labelling assays. The proportion of apoptotic cells was quantified. (B) Total‐Caspase3 and cleaved‐Caspase3 levels in C2C12 myotubes after treatment with various concentrations of LPS for 24 h. The graph represents the relative band densities. (C) C2C12 myoblasts were treated with increasing concentrations of LPS for 24–48 h. Cell viability was determined with CCK‐8 assays. (D) EdU assays were performed on C2C12 myoblasts treated with the indicated concentrations of LPS for 24 h. Representative images and quantitative analyses are shown. Each result was replicated in three independent experiments, and the data are presented as the means ±SD. *p < 0.05 versus the control group

The Annexin V‐APC/7AAD Apoptosis Detection Kit was used to clarify whether apoptosis was induced by LPS stimulation. Apoptotic cells were evaluated by flow cytometry. The proportion of apoptotic cells was less than 5% in the control groups. However, after treatment with 1 μg/ml LPS or 10 μg/ml LPS for 24 h, the percentage of apoptotic cells was increased in a dose‐dependent manner (Figure 3A). LPS also decreased the expression of total‐Caspase3 and increased the protein level of cleaved‐Caspase3 (Figure 3B). These results suggested that LPS promoted apoptosis in C2C12 myotubes.

### 3.6 LPS inhibited the proliferation of C2C12 myoblasts

The proliferation and fusion of satellite cells, which lead to an increase in the number of myonuclei, contribute to muscle growth.^29^ To explore the role of LPS in satellite cells, C2C12 myoblasts were used as a model for satellite cells. The effect of LPS on the viability and proliferation of C2C12 myoblasts was assessed via CCK‐8 and EdU assays. The results showed that the LPS‐treated C2C12 myoblasts had lower absorbances at 450 nm (Figure 3C) and lower percentages of EdU‐positive cells than the control myoblasts (Figure 3D). These results indicated that LPS inhibited the proliferation of C2C12 myoblasts.

### 3.7 LPS downregulated PLK1 expression in C2C12 cells

**FIGURE 4:**
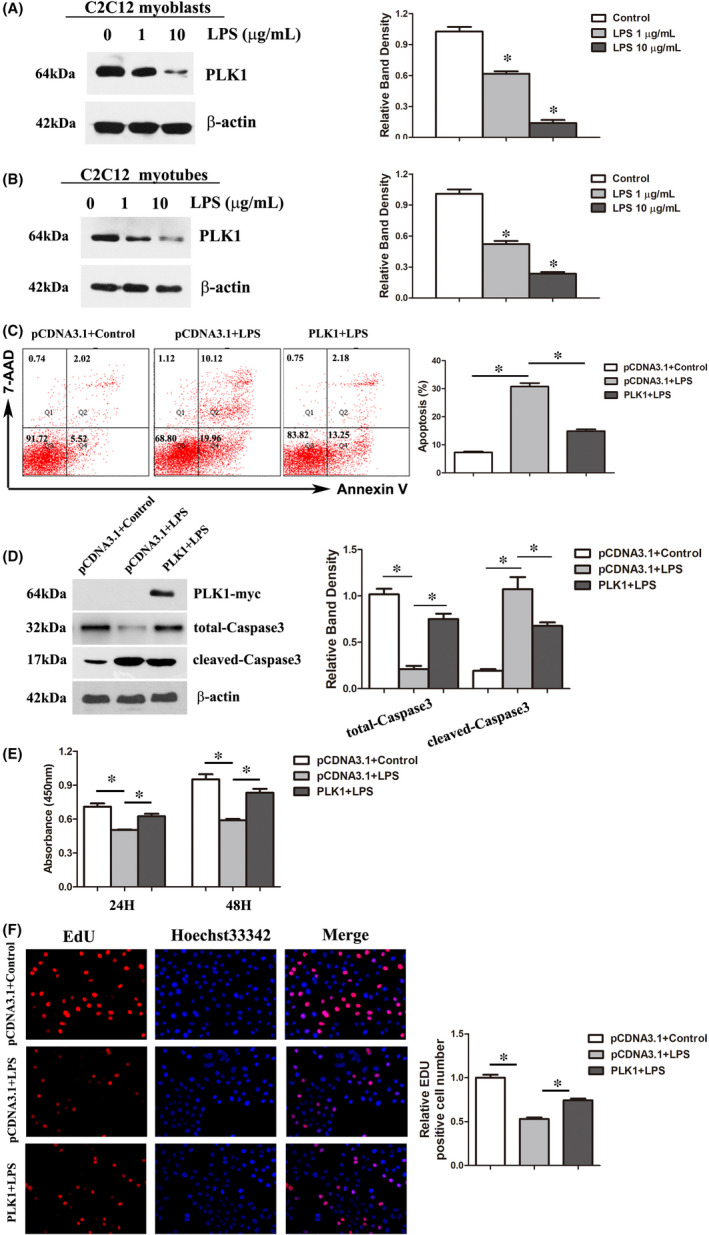
Overexpression of PLK1 attenuated LPS‐induced apoptosis and proliferation inhibition in C2C12 cells. (A‐B) The protein expression of PLK1 in C2C12 myoblasts and myotubes after treatment with various concentrations of LPS for 24 h. The graph represents the relative band densities. C2C12 cells were transfected with pcDNA‐PLK1‐myc (a PLK1 knock‐in plasmid) or the control pcDNA3.1 (the empty vector plasmid) for 24 h and then exposed to LPS (10 μg/ml) for 24 h. (C) The percentages of apoptotic C2C12 myotubes after the above treatments. (D) Levels of total‐Caspase3 and cleaved‐Caspase3 after the above treatments. (E) Cell viability was determined with CCK‐8 assays. (F) Cell proliferation assays (EdU) were performed after the above treatments. Each result was replicated in three independent experiments, and the values are the means ±SD. *p < 0.05

Since PLK1 is indispensable in the development of myogenesis, the expression of PLK1 protein was determined in LPS‐treated C2C12 myoblasts and myotubes. C2C12 cells not treated with DM were used as myoblasts. To obtain myotubes, C2C12 cells were incubated with DM for 5 days. The results showed that LPS treatment reduced PLK1 protein levels in both C2C12 myoblasts and C2C12 myotubes (Figure 4A‐B).

### 3.8 Overexpression of PLK1 attenuated LPS‐induced apoptosis and proliferation inhibition in C2C12 cells

To determine whether the lack of PLK1 contributed to LPS‐induced apoptosis and proliferation inhibition in C2C12 cells, PLK1‐overexpressing C2C12 cells were exposed to 10 μg/ml LPS for 24 h, and then, the proliferation and apoptosis of cells were assessed. We found that overexpression of PLK1 weakened the LPS effect on C2C12 proliferation and significantly decreased the number of apoptotic cells compared with the control group (Figure 4C‐F).

### 3.9 Overexpression of PLK1 partly abrogated LPS‐induced atrophy in C2C12 myotubes

**FIGURE 5:**
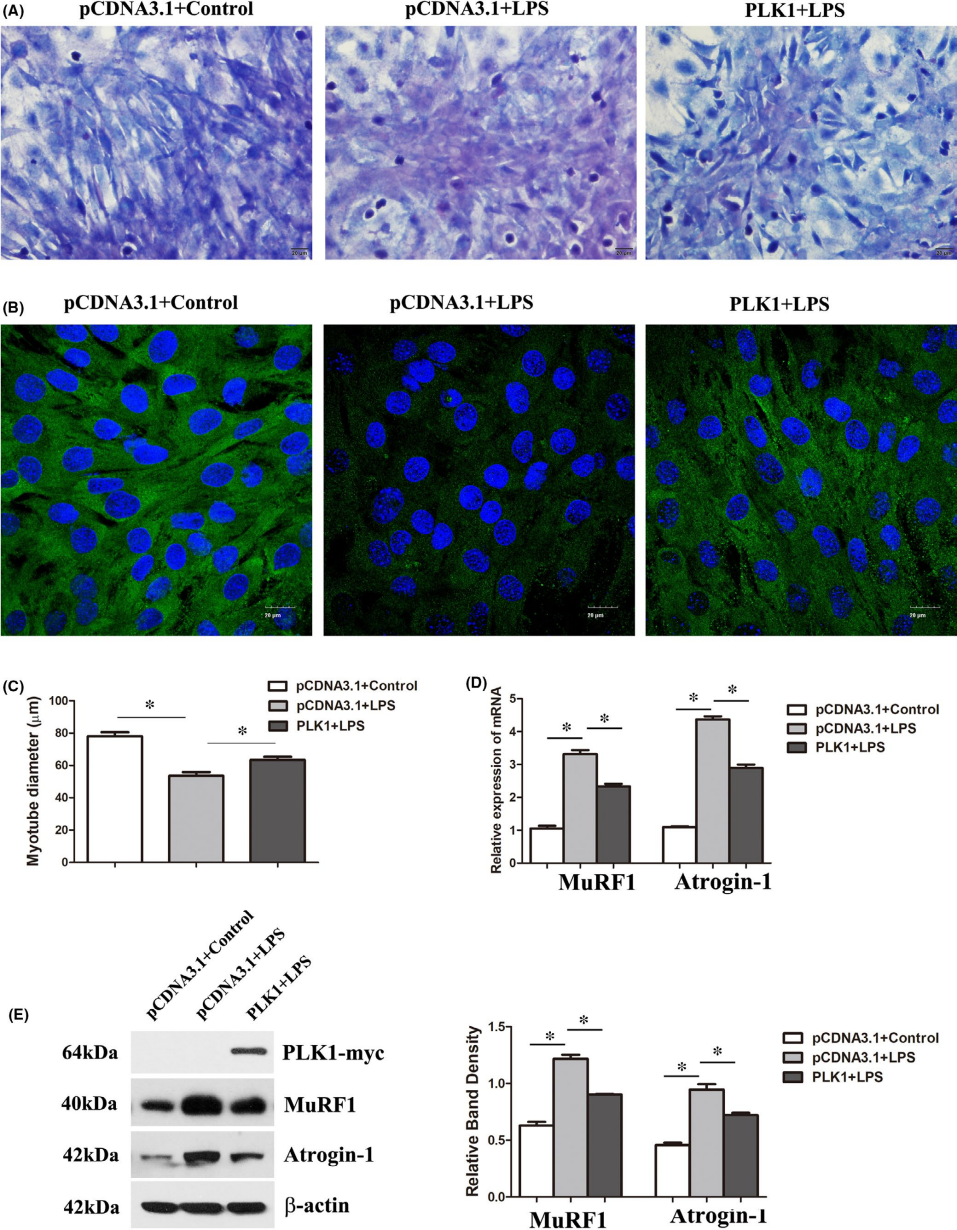
Overexpression of PLK1 partly abrogated LPS‐induced atrophy in C2C12 myotubes. Cells were transfected with pcDNA‐PLK1‐myc (a PLK1 knock‐in plasmid) or the control pcDNA3.1 (the empty vector plasmid) for 24 h and then induced to form myotubes and exposed to LPS (10 μg/ml) for 24 h. (A) Myotubes were stained with Giemsa reagent, and bright field images were taken at the same magnification. Scale bar =20 µm. (B) Representative immunofluorescence staining of MyHC in C2C12 myotubes after the above treatments. Scale bar =20 µm. (C) The diameters of the myotubes after the above treatments. (D) Quantitative RT‐PCR analysis of MuRF1 and Atrogin‐1 levels after the above treatments. (E) The protein expression levels of MuRF1 and Atrogin‐1 after the above treatments. Each result was replicated in three independent experiments, and the values are the means ±SD. *p < 0.05

Furthermore, we also explored the effect of PLK1 overexpression on LPS‐induced atrophy in C2C12 myotubes. PLK1 cDNA transfection in C2C12 myotubes increased myotube size, as indicated by morphological analysis, rescued the expression of MyHC and reduced the expression of MuRF1 and Atrogin‐1 (Figure 5A‐D), indicating that overexpression of PLK1 also partly rescued LPS‐induced atrophy in C2C12 myotubes.

### 3.10 PLK1 regulated LPS‐induced atrophy of C2C12 myotubes via the AKT pathway

**FIGURE 6:**
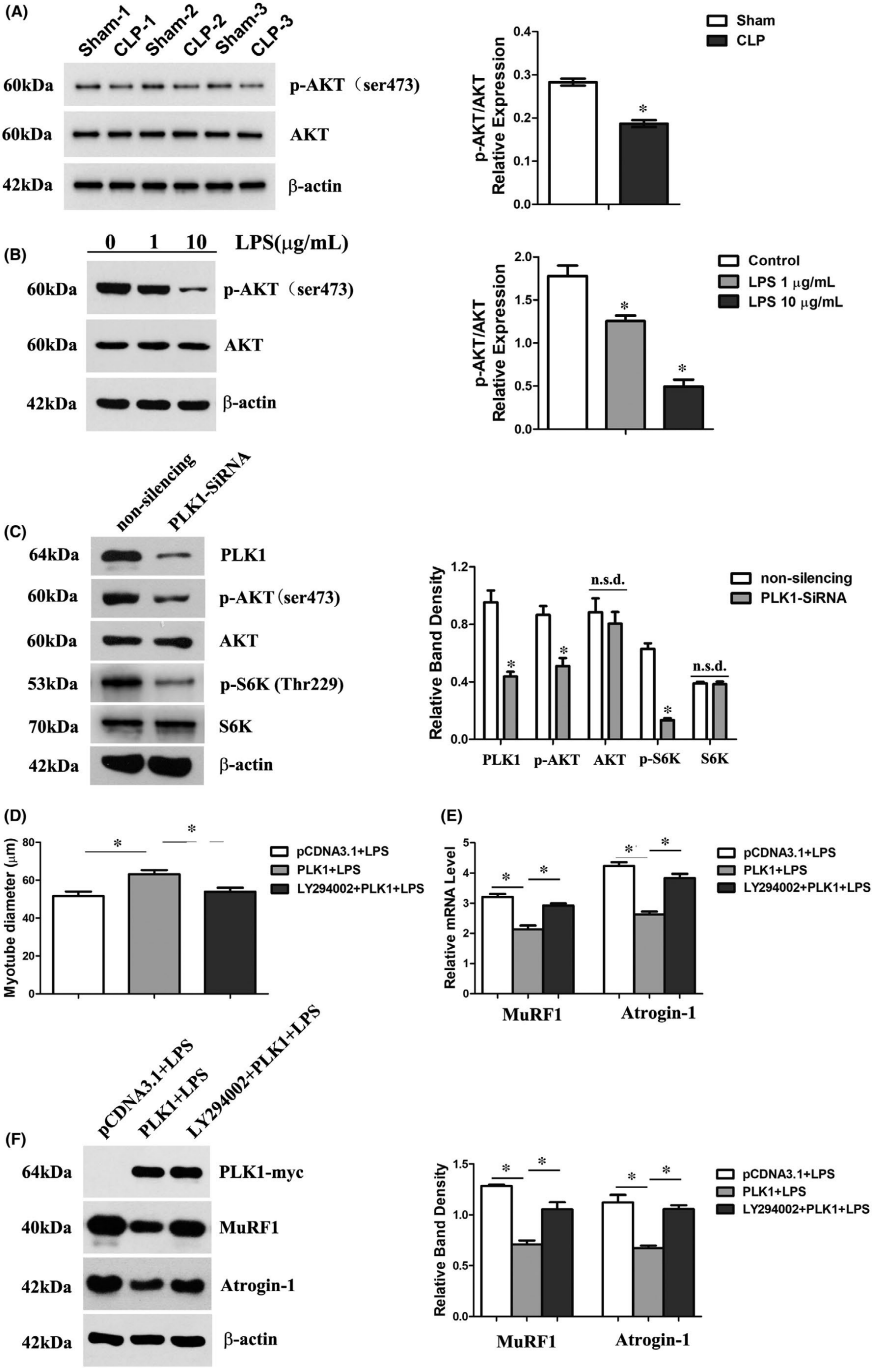
PLK1 regulated LPS‐induced atrophy of C2C12 myotubes via the AKT pathway. (A) The expression of p‐AKT in the gastrocnemius was measured 3 days after CLP. (B) The expression of p‐AKT in C2C12 myotubes after treatment with various concentrations of LPS for 24 h. (C) C2C12 cells were transfected with PLK1 siRNA or non‐silencing control siRNA and then induced to form myotubes. Cell lysates were immunoblotted for the indicated proteins. (D) PLK1‐overexpressing C2C12 myotubes were pre‐treated with LY294002 (an inhibitor of the AKT pathway), and then, the diameters of the myotubes were measured. (E) The mRNA levels of MuRF1 and Atrogin‐1 after the above treatments. (F) The protein expression levels of MuRF1 and Atrogin‐1 after the above treatments. Each result was replicated in three independent experiments, and the values are the means ±SD. *p < 0.05

Since the AKT pathway was reported to contribute to the prevention of muscle atrophy and our previous study suggested that PLK1 acts as an upstream regulator of AKT,^25^, ^30^ we assumed that PLK1 rescued LPS‐induced atrophy of C2C12 myotubes via the AKT pathway. To verify this hypothesis, we first evaluated the activity of AKT in myofibers during sepsis, and the results showed that sepsis induced downregulation of p‐AKT expression in vivo and in vitro (Figure 6A‐B). Moreover, measurement of the activity of AKT in PLK1‐depleted C2C12 cells revealed reductions in the expression of p‐AKT and its downstream protein p‐S6K (Figure 6C). Furthermore, LY294002, an inhibitor of the AKT pathway, was pre‐treated in PLK1‐overexpressing C2C12 myotubes followed by incubation with LPS. The results revealed that inhibition of AKT deteriorated LPS‐induced atrophy in C2C12 myotubes overexpressing PLK1 (Figure 6D‐F).

### 3.11 PLK1 participated in regulating the apoptosis pathway and E3 ubiquitin ligase

**FIGURE 7:**
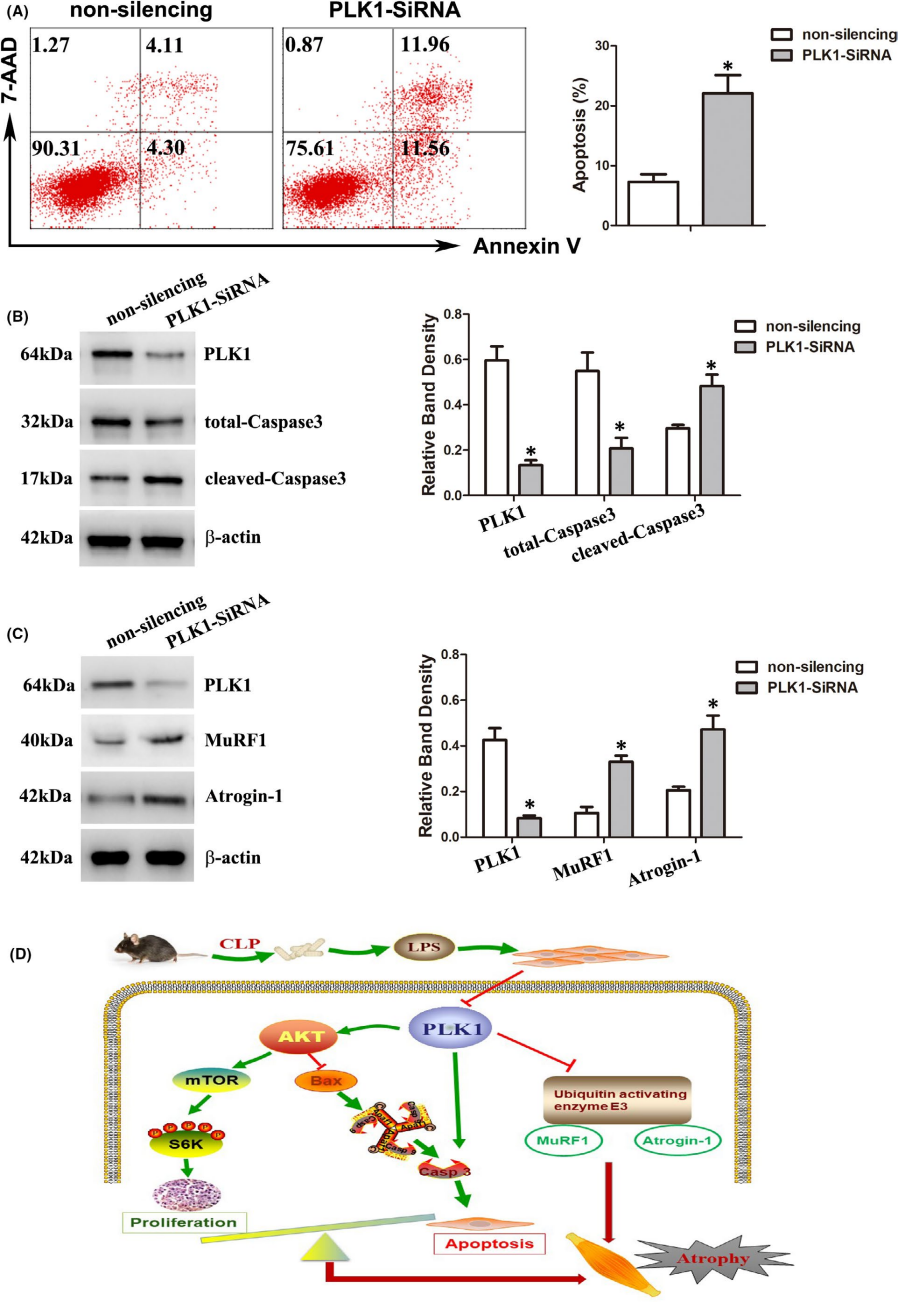
PLK1 participated in regulating the apoptosis pathway and E3 ubiquitin ligases. (A) C2C12 cells were transfected with PLK1 siRNA or non‐silencing control siRNA and then induced to form myotubes. Apoptosis was analysed by Annexin V‐FITC/7‐AAD double‐labelling assays. The proportion of apoptotic cells was quantified. (B) Total‐Caspase3 and cleaved‐Caspase3 levels were measured after the above treatments. The graph shows the relative band densities. (C) The expression levels of the indicated proteins were measured after the above treatments. The graph shows the relative band densities. Each result was replicated in three independent experiments, and the values are the means ±SD. *p < 0.05. (D) Diagram representation of PLK1‐AKT signalling in sepsis‐induced muscle atrophy. Sepsis/LPS induces the downregulation of PLK1 expression in myofibres, thus reducing the activity of the AKT pathway. This reduction results in apoptosis of myotubes and inhibits the proliferation of myoblasts, ultimately leading to myofibre atrophy. Meanwhile, PLK1 also targets the Caspase3‐dependent apoptosis pathway and inhibits the activity of the E3 ubiquitin ligases MURF1 and Atrogin‐1, which may be another mechanism of sepsis‐induced muscle atrophy

As apoptosis and the ubiquitin‐proteasome pathway play critical roles in skeletal muscle atrophy, we then explored the effect of PLK1 on apoptosis and the activity of the typical E3 ubiquitin ligases MuRF1 and Atrogin‐1. Transfection of C2C12 cells with PLK1 siRNA increased the percentage of apoptotic cells, as measured by flow cytometry; decreased total‐Caspase3 expression; and increased cleaved‐Caspase3 expression (Figure 7A‐B). In addition, the expression of MuRF1 and Atrogin‐1 was increased in PLK1‐depleted C2C12 cells, indicating ubiquitin ligase enzyme activation (Figure 7C).

## 4 DISCUSSION

In the present study, we used in vivo and in vitro sepsis models to investigate the mechanism of sepsis‐induced skeletal muscle atrophy. Sepsis downregulated the expression of PLK1 in muscle cells and subsequently inhibited the activity of the AKT pathway, leading to apoptosis of myofibrils and a reduction in myoblast proliferation and thereby resulted in muscle atrophy. PLK1 also targeted the Caspase3‐dependent apoptosis pathway and E3 ubiquitin ligases, which may be another mechanism of sepsis‐induced muscle atrophy (Figure 7D).

Sepsis‐induced skeletal muscle atrophy is the result of a series of factors, including inflammation and metabolic dysfunction. Here, we illustrated that PLK1 plays a critical role in the pathological process. PLK1 is the most extensively characterized member of the PLK family, which is composed of serine/threonine protein kinases and includes five members (PLK1, PLK2, PLK3, PLK4 and PLK5).^31^ Previous studies have proven that inhibition of PLK1 expression leads to prolonged G2/M arrest, mitotic spindle defects and mitotic catastrophe, thus sensitizing cells to apoptosis.^25^, ^32^ Apoptosis is a process of programmed cell death that is important for maintaining tissue homeostasis in humans. However, excessive apoptosis in myofibres can lead to the onset and progression of muscle atrophy.^33^ The current results revealed that the number of TUNEL‐positive nuclei in the gastrocnemius was increased significantly in septic mice and that the proportion of apoptotic cells was increased in LPS‐treated C2C12 myotubes. At the same time, Caspase3, which acts as a regulator of mitochondria‐mediated apoptosis, was activated. Furthermore, downregulation of PLK1 with siRNA contributed to an increase in the proportion of apoptotic C2C12 cells. The results also verified the protective role of PLK1 against apoptosis, which is in accordance with its functions as a pleiotropic master regulator of mitosis and coordinator of DNA replication after stress.^34^

The development of muscle requires myoblasts to undergo myogenesis and form myofibres. The process includes proliferation, migration, differentiation and fusion, among which the proliferation of myoblasts is critical in muscle regeneration.^35^ The classic PI3K/AKT signalling pathway plays an important role in regulating cell growth, survival, proliferation and cell cycle progression.^36^ AKT is a downstream target gene of PI3K, and its activation is followed by activation of the mTOR/S6K/4EBP1 pathway, which promotes protein synthesis through increases in translation initiation and elongation. Activated AKT suppresses apoptosis by phosphorylating the Bcl‐2 family member Bad and disrupting its ability to induce cell death, thus promoting cell survival.^37^ In our study, LPS promoted apoptosis of myotubes and inhibited proliferation of myoblasts, thus reducing muscle mass and suppressing myofibre regeneration and ultimately resulting in muscle atrophy. During the atrophy process, the activity of AKT was suppressed, indicating the critical role of AKT in regulating apoptosis and proliferation of C2C12 cells.

The relationship between PLK1 and AKT is still under debate. Zhang reported that PLK1 activated the PI3K/AKT/mTOR pathway by phosphorylating PTEN (Ser385) during oxidative stress, while another study showed that PI3K/Akt‐dependent phosphorylation of PLK1 (Ser99) is required for metaphase‐anaphase transition.^20^, ^38^ In the present study, we found that depletion of PLK1 caused a reduction in the levels of phospho‐AKT (Ser473), suggesting that PLK1 may act upstream of the AKT pathway. Furthermore, inhibiting the AKT pathway deteriorated LPS‐induced atrophy in C2C12 myotubes overexpressing PLK1, indicating that PLK1 protects against LPS‐induced atrophy of C2C12 myotubes via the AKT pathway.

The UPS has been demonstrated to play a crucial role in mediating muscle atrophy by modulating the different processes that determine muscle mass, such as myogenesis, protein synthesis and degradation.^39^ The ubiquitination process depends on three groups of enzymes termed E1 (activating) enzymes, E2 (conjugating) enzymes and E3 ligases. MuRF1 and Atrogin‐1 act as typical E3 ubiquitin ligases in muscle atrophy. Recently, inhibition of MURF1 and Atrogin‐1 has been considered an effective method of muscle atrophy treatment in different models because it reduces protein degeneration through the UPS.^40^ In this study, inhibition of PLK1 resulted in upregulation of the expression of MURF1 and Atrogin‐1, suggesting that PLK1 may act upstream of E3 ubiquitin ligases.

Several limitations of the present study should be noted. Primarily, sepsis‐induced skeletal muscle atrophy is a complicated pathological process, and no suitable in vitro model has been designed to replicate this sepsis state. Therefore, C2C12 cells were used in in vitro studies to explore the effects of LPS on the apoptosis, proliferation and atrophy of C2C12 cells. Moreover, the atrophy of myotubes following LPS treatment could not completely recovered by targeting the PLK1‐AKT pathway, indicating that other mechanisms exist in the atrophy process, and the relationship of PLK1 with the whole UPS deserves further exploration. In addition, in evaluating AKT activity, we only assessed the expression of p‐AKT (Ser473), which was extensively detected.

In summary, our study demonstrates that sepsis induces skeletal muscle atrophy by promoting apoptosis of myofibres and inhibiting proliferation of myoblasts and that this process is regulated by the PLK1‐AKT pathway. PLK1 also regulates the Caspase3‐dependent apoptosis pathway and the activity of the E3 ubiquitin ligases MURF1 and Atrogin‐1, which may be another mechanism of sepsis‐induced myofibre atrophy. These findings enhance understanding of the mechanism of sepsis‐induced skeletal muscle atrophy and provide evidence that the PLK1‐AKT pathway may be a novel therapeutic target to relieve skeletal muscle atrophy induced by sepsis.

## CONFLICT OF INTEREST

All authors declare no conflicts of interest regarding this paper.

## AUTHOR CONTRIBUTIONS

**Ying‐Ya Cao:** Funding acquisition (equal); Writing‐review & editing (equal). **Zhen Wang:** Methodology (equal); Writing‐review & editing (equal). **Tao Yu:** Resources (equal). **Yuan Zhang:** Methodology (equal). **Zhong‐Han Wang:** Methodology (equal). **Zi‐Meng Lu:** Methodology (equal); Software (equal). **Wei‐Hua Lu:** Funding acquisition (equal); Supervision (equal). **Jian‐Bo Yu:** Supervision (equal).

## Supporting information

Supplementary Material
