# Supplementary material for: Sepsis induces muscle atrophy by inhibiting proliferation and promoting apoptosis via PLK1‐AKT signalling
Source: J Cell Mol Med. 2021 Sep 12;25(20):9724–39. doi: 10.1111/jcmm.16921 (PMC8505846; doi:10.1111/jcmm.16921)
Supplement: Supplementary file 1 — Supplementary Material [file JCMM-25-9724-s001.pdf]

SUPPLEMENTARY FILES TO

Sepsis Induces Muscle Atrophy by Inhibiting Proliferation and Promoting Apoptosis Via PLK1-AKT Signalling

Ying-Ya Cao[1], Zhen Wang[2], Tao Yu[3], Yuan Zhang[1], Zhong-Han Wang[2],Zi-Meng Lu[4], Wei-Hua Lu[2]*, Jian-Bo Yu[1]*

1. Department of Anesthesiology and Critical Care Medicine, Tianjin Nankai Hospital, Tianjin Medical University, Tianjin, 300100, China;

2. Department of Intensive Care Unit, The First Affiliated Hospital of Wannan Medical College, Wuhu 241001, Anhui, China;

3. Department of Neurosurgery, The First Affiliated Hospital of Wannan Medical College, Wuhu 241001, Anhui, China;

4. College of Food Science and Engineering, Northwest A&F University

Ying-Ya Cao and Zhen Wang contributed equally to this work

Corresponding authors: Jian-Bo Yu (Email: 30717008@nankai.edu.cn) or

Wei-Hua Lu (E-mail: lwh683@126.com)

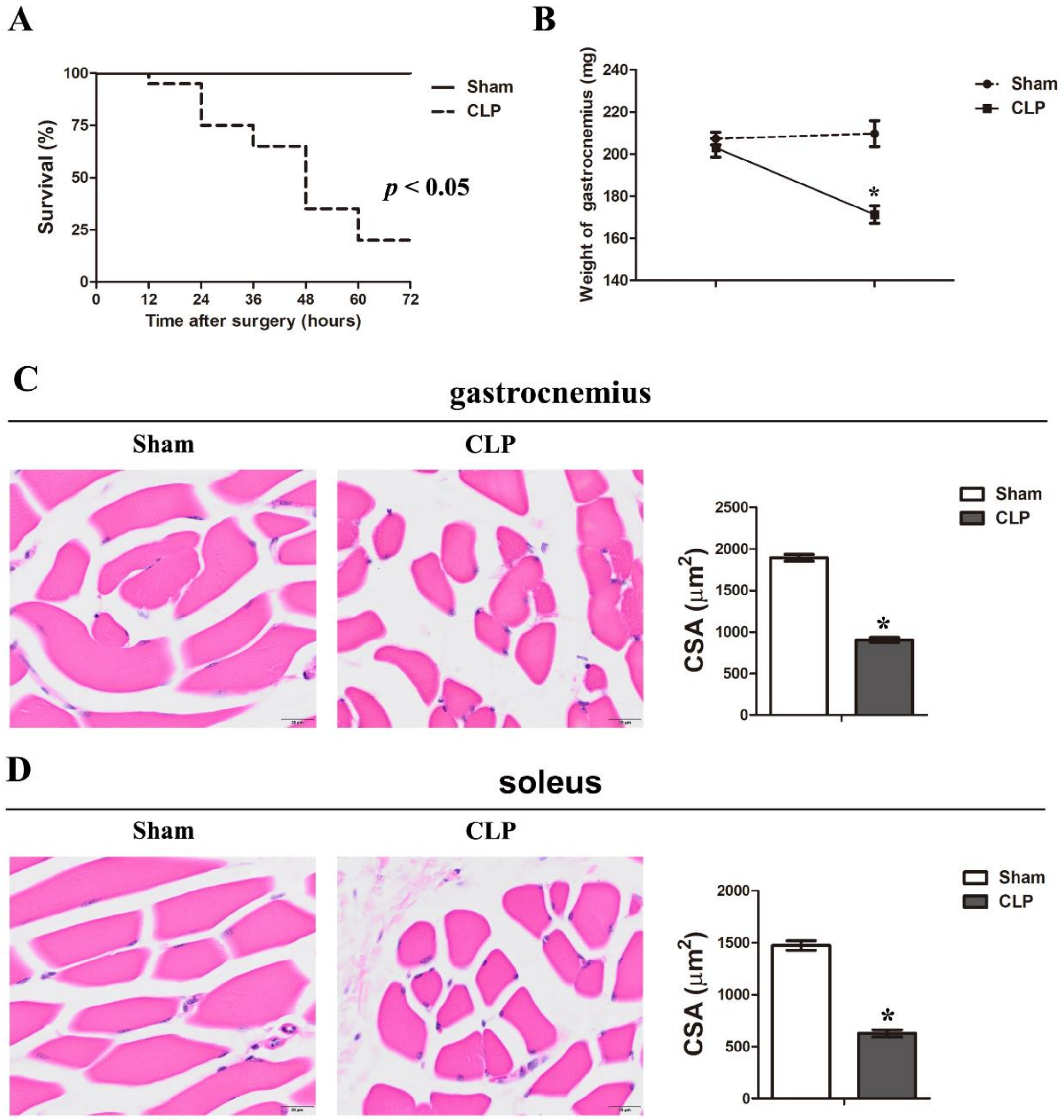

Supplementary Figure 1 (Fig. S1) Sepsis induced atrophy of skeletal muscles of mice. Mice were subjected to CLP to establish a sepsis model and were sacrificed 3 days later. (A) After CLP or sham laparotomy, the mice were observed every 12 h until 72 h to record the survival ratio in the two groups (log-rank test, $P < 0.05$). (B) Gastrocnemius weight for the mice in the two groups. (C) Typical H&E-stained sections and quantification of the CSA of the gastrocnemius in the two groups (bar = 20 μm; magnification =400×). (D) Typical H&E-stained sections and quantification of the CSA of the soleus in the two groups (bar = 20 μm; magnification =400×). *$P < 0.05$.

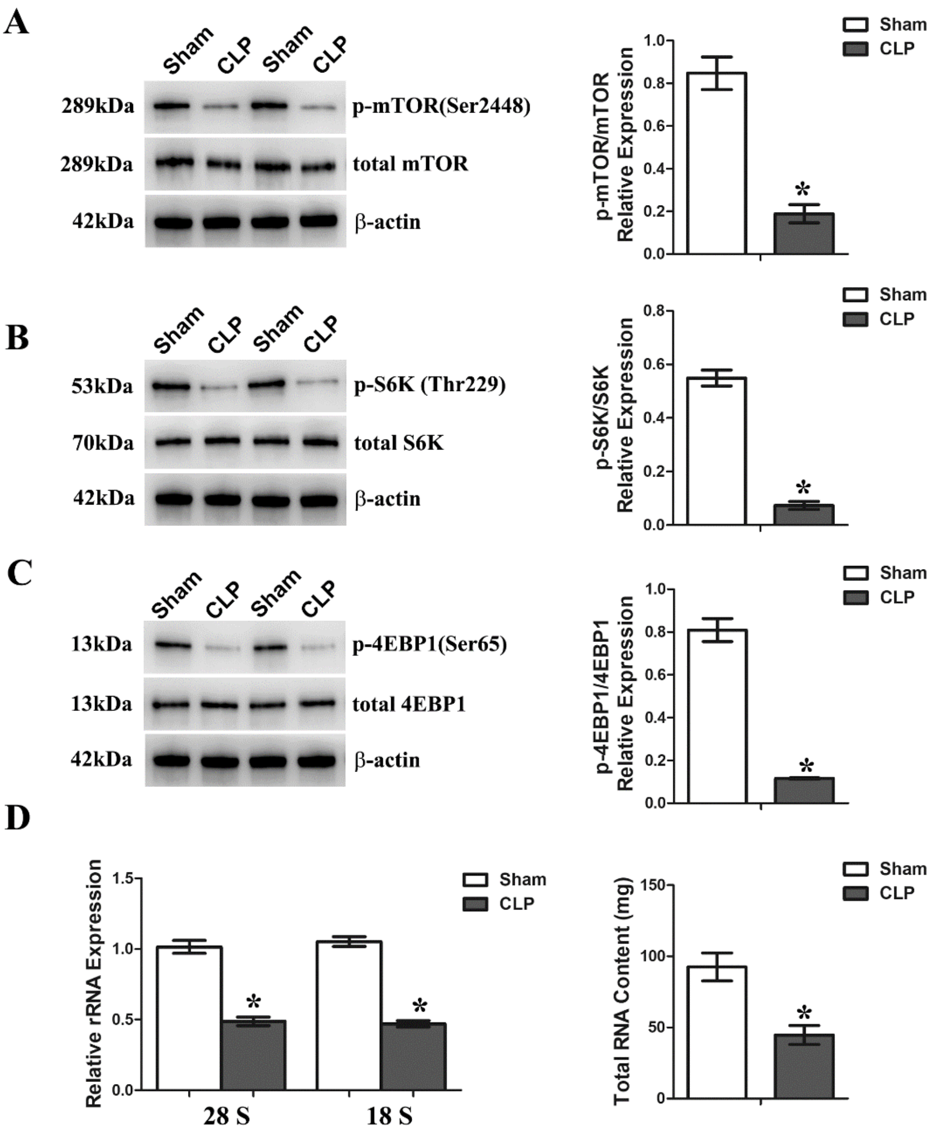

Supplementary Figure 2 (Fig. S2) Sepsis inhibited the protein synthesis of skeletal muscles in mice. Mice were subjected to CLP to establish a sepsis model and were sacrificed 3 days later. (A-C) The expression levels of the indicated proteins in gastrocnemius muscle were measured after the above treatments. The graph shows the relative band densities. (D) Expression of rRNAs (28S, 18S) in gastrocnemius muscle. (E) Total RNA content in gastrocnemius muscle. Each result was replicated in three independent experiments, and the values are the means ± SD (n= 3). *$P < 0.05$.
